# P-1720. Histopathological Assessment as a Cornerstone in the Diagnosis of Invasive Fungal Infections: A 10-Year Experience in a Tertiary-Level Hospital in Mexico

**DOI:** 10.1093/ofid/ofaf695.1891

**Published:** 2026-01-11

**Authors:** Luis Felipe Arias-Ruiz, Griselda Medina-Montaño, Sandra Guzmán-Tepetla, Diego Alberto Conde-Gutiérrez, Rita Dorantes-Heredia, Daniel Aguilar-Zapata

**Affiliations:** Hospital Médica Sur, Mexico City, Distrito Federal, Mexico; Medica Sur, Tlalpan, Distrito Federal, Mexico; Hospital Médica Sur, Mexico City, Distrito Federal, Mexico; Hospital Médica Sur, Mexico City, Distrito Federal, Mexico; Hospital Médica Sur, Mexico City, Distrito Federal, Mexico; Hospital Medica Sur, Mexico City, Mexico City, Mexico

## Abstract

**Background:**

The main objective is to highlight the critical role of histopathological examination in the identification of invasive fungal infections from 10 year samples in our hospital in Mexico City.

**Methods:**

A retrospective, descriptive and epidemiological cohort was conducted at Medica Sur Hospital from January 2014 to December 2024. Data regarding comorbidities, particularly immunosuppressive conditions, microbiological findings (culture), serological tests, and whether or not treatment was administered, was collected and analyzed.

**Results:**

We collected a total of 143 samples related to any fungal morphology, however, we excluded yeast from saprophyte tissues and colonizers molds. We included 42 samples that were considered as invasive fungal infections. *Histoplasma* was the most frequent pathogen (28.6%), 50% of those samples were lung biopsies. Clinically 50% of those patients with *Histoplasma* were under immunosuppressive therapy. 75% of these patients received antifungal therapy. *Pneumocystis* was the second most common identified fungus (21.4%), 100% coming from lung biopsies. The baseline diseases that predominated with *Pneumocystis* pneumonia were patients living with HIV, patients taking immunosuppressive medications and immunocompetent patients, with 44% each. *Aspergillus* was identified in 17%, with a lung biopsy predominance in 43% of the cases. We had 50% of correlation with positive *Aspergillus* cultures. Only 29% of these patients received antifungal treatment. *Mucor* was the fourth most common fungus (12%); 20% of *Mucor* patients correlated with a positive culture. In 80% of *Mucor* cases the patients were under immunosuppressive treatment. *Cryptococcus* (9.5%) was identified most frequently in lung biopsies (50%), and 50% of these patients were immunocompetent. *Coccidioides* (5%), and *Blastomyces* (5%) were identified in lung biopsies (100%). Finally, *Fusarium* represented 2% of the cases, identified in a patient under immunosuppressive medications; the patient received antifungal treatment.

**Conclusion:**

The morphological description of fungal elements in tissue is essential for the diagnosis of invasive fungal infections, which guides decision-making in the clinical setting for an adequate treatment.

**Disclosures:**

All Authors: No reported disclosures

